# The Bivalent Rewarding and Aversive properties of Δ^9^-tetrahydrocannabinol are Mediated Through Dissociable Opioid Receptor Substrates and Neuronal Modulation Mechanisms in Distinct Striatal Sub-Regions

**DOI:** 10.1038/s41598-019-46215-7

**Published:** 2019-07-05

**Authors:** Christopher Norris, Hanna J. Szkudlarek, Brian Pereira, Walter Rushlow, Steven R. Laviolette

**Affiliations:** 1 Addiction Research Group, Schulich School of Medicine & Dentistry, University of Western Ontario, London, Ontario N6A 5C1 Canada; 2 Dept. of Anatomy & Cell Biology, Schulich School of Medicine & Dentistry, University of Western Ontario, London, Ontario N6A 5C1 Canada; 3 Dept. of Psychiatry, Schulich School of Medicine & Dentistry, University of Western Ontario, London, Ontario N6A 5C1 Canada

**Keywords:** Motivation, Reward

## Abstract

The primary psychoactive compound in cannabis, Δ^9^-tetrahydrocannabinol (THC), is capable of producing bivalent rewarding and aversive affective states through interactions with the mesolimbic system. However, the precise mechanisms underlying the dissociable effects of THC are not currently understood. In the present study, we identify anatomically dissociable effects of THC within the rat nucleus accumbens (NAc), using an integrative combination of behavioral pharmacology and *in vivo neuronal* electrophysiology. We report that the rewarding vs. aversive stimulus properties of THC are both anatomically and pharmacologically dissociable within distinct anterior vs. posterior sub-regions of the NAc. While the rewarding effects of THC were dependent upon local μ-opioid receptor signaling, the aversive effects of THC were processed via a κ-opioid receptor substrate. Behaviorally, THC in the posterior NASh induced deficits in social reward and cognition whereas THC in the anterior NAc, potentiated opioid-related reward salience. *In vivo* neuronal recordings demonstrated that THC decreased medium spiny neuron (MSN) activity in the anterior NAc and increased the power of gamma (γ) oscillations. In contrast, THC increased MSN activity states in the posterior NASh and decreased γ-oscillation power. These findings reveal critical new insights into the bi-directional neuronal and pharmacological mechanisms controlling the dissociable effects of THC in mesolimbic-mediated affective processing.

## Introduction

∆^9^-tetrahydrocannabinol (THC), the primary psychoactive component of cannabis, strongly modulates affective processing via interactions with the mesolimbic circuitry. THC can produce both rewarding and aversive effects in humans and other animals^1–4^ and strongly modulates the mesolimbic dopamine (DA) system. For example, acute THC administration in the rodent NAc strongly activates ventral tegmental area (VTA) DAergic neuronal activity^5^ and systemic THC administration directly activates VTA DA neurons^6^. In humans, chronic cannabis use has been shown to induce salience sensitization to cues associated with cannabis administration, via sensitization of the mesolimbic system^7^. Nevertheless, the precise neuroanatomical and pharmacological mechanisms by which THC may produce rewarding, dependence-producing effects vs. neuropsychiatric side-effects, are not currently understood.

The primary pharmacological target of THC, CB_1_ receptors (CB1R), are widely distributed throughout the brain, including the mesolimbic circuitry. Substantial evidence implicates the nucleus accumbens (NAc) as an important area for THC’s effects on cognitive and affective processing. For example, systemic THC causes opioid receptor (OR)- dependent DA efflux in the NAc^3,8^ and chronic THC exposure alters synaptic plasticity within the NAc^9^. Importantly, the NAc is involved in both reward and aversion processing, particularly the medial shell subregion (NASh), which is implicated in motivational salience, the processing of emotional stimuli, reinforcement, and addiction^10–13^.

The accumbens is a highly complex limbic structure involved in processing both reward and aversion-related behaviors. Previous evidence has identified the anterior pole of the NASh as a region containing high concentrations of µ-opioid receptors^14^. Thus, stimulation of the anterior NASh with a µ-opioid receptor (µOR) agonist produces reward while stimulation of the posterior NASh with a κ-opioid (κOR) agonist, produces aversion^15^. In addition, there is significant co-localization of CB1Rs and ORs within the NASh region^16^. However, how an exogenous cannabinoid like THC might influence striatal affective processing through CB1-OR signaling remains unknown. Given that THC serves as a partial CB1 receptor agonist, this evidence may suggest that the differential affective stimulus effects of THC might depends upon differential activation of anatomically distinct OR subtypes, within the mammalian NAc.

At the neuronal level, medium spiny neuron (MSN) activity states are functionally linked to reward and aversion processing, with reward states characterized by decreased MSN activity levels, and aversive states associated with increased activity^17,18^. Additionally, neuronal population activity within the NAc, specifically γ-oscillations, are associated with the processing of reward-related information^19^ and disturbances in γ-oscillations are linked to various neuropsychiatric disturbances, including schizophrenia-related affective dysregulation^20^.

In the present study, we investigated the effects of direct microinfusions of THC on reward and aversion conditioning, social behavior and neuronal activity states within the NASh. Using a combination of behavioral assays and *in vivo* neuronal electrophysiology, we report that THC infused into the anterior NASh produces µ-opioid receptor dependent reward, potentiates morphine reward salience, decreases medium spiny neuron activity and increases the power of high frequency γ-oscillations. In contrast, THC in the posterior NASh produces κOR dependent aversion, impairs social recognition, increases medium spiny neuron activity and decreases the power of high frequency γ-oscillations in local field potential. These findings reveal novel dissociable and distinct mechanisms for the bivalent motivational effects of THC directly in the NAc.

## Materials and Methods

### Animals and surgery

Male Sprague Dawley rats (300 to 350 g; *n* = 204) were obtained from Charles River (Quebec, Canada). Rats were excluded from analysis if histological analysis showed the placement of the cannula outside of the intended area (*n* = 19). Rats were housed under controlled conditions (12-hour light/dark cycle and food/water access ad libitum). All procedures were performed in accordance with Governmental and Institutional guidelines for appropriate rat care and experimentation. The experimental protocols were approved by the Canadian Council on Animal Care and the Animal Care Committee at Western University, Ontario. Rats were anesthetized with a mixture of ketamine (80 mg/ml) and xylazine (6 mg/ml) and placed in a stereotaxic device. Stainless steel guide cannula (22 gauge; PlasticsOne) were implanted bilaterally into either the aNASh at the following coordinates^21^: anteroposterior (AP): +2.5 mm from bregma, lateral (L): ±2.6 mm, dorsal-ventral (DV): −7.4 mm from the dural surface, or the pNASh at the following coordinates: (AP): +1.5 mm from bregma, (L): ±2.6 mm, (DV): −7.4. Guide cannulae were held in place using jeweler’s screws and dental acrylic. Rats were then single housed following recovery. After completion of behavioral experiments, rats received an overdose of pentobarbital (240 mg/kg, i.p.) and were perfused with isotonic saline followed by 10% formalin. Brains were extracted and post-fixed 24 hrs before being placed in a 25% formalin-sucrose solution for one week. Brains were sliced (60 μm) using a cryostat and stained with Cresyl violet. Injector tips placements were localized using a light microscope. Rats with cannula placements found outside the anatomical boundaries of the NASh were excluded from data analysis.

### Drug administration

THC was received dissolved in ethanol. The solution was mixed with cremaphor and the ethanol was evaporated with a nitrogen stream. Physiological saline was then added to achieve the desired concentrations. Cyprodime was first dissolved in DMSO and diluted with physiological saline to the desired concentration. Nor-BNI was dissolved in warmed physiological saline. Individual vehicle solutions were also prepared as control for each condition. Rats received intra-NASh microinfusions with either THC (Cayman Chemical, 10–100 ng/0.5 µl), cyprodime (CYP; Tocris; 500 ng or 1 µg/0.5 µl), *nor*-binaltorphimine dihydrochloride (*nor*-BNI; Tocris; 500 ng or 1 µg/0.5 µl), THC co-administered with CYP, or THC co-administered with *nor*-BNI, immediately prior to conditioning. Intra-NASh drugs were delivered via a 28-gauge microinfusion syringe over a period of 1 minute. Microinjectors were left in place for an additional 1 min to ensure adequate diffusion from the tip. All intracerebral infusions were 0.5 μL total volume per side. Previous work has demonstrated that at this volume, diffusion outside of the targeted area is low^15^. Morphine sulfate (Macfarland-Smith) was administered i.p. The intra-cranial dose selections for all of the above compounds were based upon our previously published or piloted dose-response curves using these compounds^22,23^ which have demonstrated maximal behavioral efficacy and the absence of non-specific behavioural and/or off-site pharmacological side effects. For morphine CPP experiments, rats were injected intra-peritoneally with either morphine (0.05 mg/kg) or saline on alternating conditioning days.

### Conditioned place preference

All rats were conditioned using an unbiased, fully counterbalanced place conditioning procedure as described previously (Ahmad *et al*.^22^; Laviolette and van der Kooy^24^). The two conditioning environments differed in smell, texture and color. One environment was black, with a smooth Plexiglass floor wiped down with 2% acetic acid prior to each conditioning session. The other environment was white, with a wire mesh floor covered with woodchips. Prior to commencement of CPP conditioning, rats are given a preconditioning phase where they are placed into a motivationally neutral gray box for 20 min, 24 h prior to start of conditioning. CPP conditioning consists of 4 drug-environment and 4 vehicle-environment pairings once per day for 30 min each session, alternating over an 8-day period. Environmental conditioning exposures are fully counterbalanced for both environment assignment and drug/vehicle presentations. We have demonstrated previously that these conditioning environments are motivationally neutral at the beginning of experiments such that naïve rats do not show a significant preference or aversion for either environment prior to the onset of conditioning^24^. During the CPP test phase, rats are placed in a neutral gray zone separating the drug and vehicle environments and allowed to move freely for a period of 10 min between environments. Times spent in each environment are digitally recorded and analyzed offline. All rats are tested in a drug-free state. For experiments examining the effects of intra-NASh THC and/or opioid receptor challenges, experimental groups received intra-NASh microinfusions of either THC (10–100 ng/0.5 µl), cyprodime (500 ng–1 µg/0.5 µl), *nor*-BNI (500 ng–1 µg/0.5 µl), THC co-administered with cyprodime, or THC co-administered with *nor*-BNI vs. VEH microinfusions. A subsequent CPP experiment examined the potential effects of intra-NASh THC on morphine reward salience, using a sub-reward threshold conditioning dose of morphine (0.05 mg/kg, i.p.) which we have previously reported does not produce significant morphine CPP in and of itself (Ahmad *et al*.^22^; Ahmad and Laviolette^25^; Loureiro *et al*.^26^). For these experiments, THC or VEH control groups received either anterior or posterior THC or VEH microinfusions before being injected with either sub-threshold morphine (0.05 mg/kg) or saline on alternating conditioning days. Thus, the potential motivational effects of intra-NASh THC (rewarding or aversive) were balanced across both saline and morphine conditioning environments.

### Sucrose preference test

Prior to testing, rats were water deprived for 12 hrs. At testing, rats were given two bottles of a 2% sucrose solution to drink for 12 hrs. After exposure to the sucrose solution, rats were micro-infused with either THC (100 ng/0.5 µl) or vehicle. They were then placed back in their cages and given access to one bottle of regular water or one bottle of 2% sucrose solution. A sucrose preference index was calculated for each rat and averaged across groups by taking the percentage of the volume of sucrose intake over the total volume of fluid intake over the 60-min test phase.

### Sociability and social memory

Testing was performed in a rectangular, three-chambered box. For the sociability test, rats were placed in the middle chamber for 5 minutes. Following habituation, an unfamiliar male rat was placed in one of the side chambers in a rectangular plexiglass cage. The location of the rat was counterbalanced between subjects. The subject was then infused with either THC (100 ng/0.5 µl) or vehicle in either their aNASh or pNASh. The subject was then allowed to explore the entire apparatus for 8 minutes. Entries were defined as all four paws present in one chamber. Behavioral performance was expressed using sociability scores (i.e., difference between times spent in stranger vs. empty compartments). Next, to evaluate social recognition, each rat was tested in an 8-minute session to evaluate social memory. A second, unfamiliar rat was placed in the previously empty chamber. The test rat had a choice between the previously encountered rat versus the novel, unfamiliar rat. Times spent in each chamber were recorded, and a social recognition score (i.e., difference between times spent in the nonfamiliar vs. familiar rat chamber) was calculated for each rat. Times spent in each chamber were recorded with a video-tracking system (ANY- maze) during all tests. A sociability (time spent with the other rat vs. time spent with the empty cage) and social recognition score (time spend with the novel rat vs. time spent with the novel rat) was calculated for each rat.

### *In Vivo* electrophysiological recordings

*In vivo* extracellular recordings were performed as described previously^26–28^. Rats were anesthetized with urethane (1.4 g/kg, i.p.) and placed in a stereotaxic apparatus with body temperature maintained at 37 °C. A scalp incision was made to remove the skin above the skull, and holes were drilled in the skull above the NASh and the cranial ventricle. For intra-cranial ventricle (ICV) microinfusions of THC (1 µg/μL), a 10 μL gastight Hamilton syringe was slowly lowered into the cranial ventricle (15˚ angle): AP: −0.9 mm from bregma, LAT ± 2.7 mm, DV: −3.8 mm from the dural surface. For intra-NASh extracellular recording, glass micro-electrodes (with an average impedance of 6 to 8 MΩ) filled with a 2% Pontamine Sky Blue solution were lowered using a hydraulic micro-positioner (Kopf 640) at the following flat skull stereotaxic coordinates: AP: +1.5 or +2.5 mm from bregma, LAT: ±0.8 mm, DV: −6.0 to −8.0 mm from the dural surface. Extracellular signals were amplified using a MultiClamp 700B amplifier (Molecular Devices) and recorded through a Digidata 1440A acquisition system (Molecular Devices) using pClamp 10 software. Extracellular recordings were filtered at 1 kHz and sampled at 5 kHz. NASh medium spiny neurons were identified using previously established criteria. Any cells with a spike width of less than 1 ms and more than 2 ms were excluded from analysis. The electrode was used to simultaneously record local field potentials (LFP). Recording analyses were performed with Clampfit 10 software. Response patterns of isolated NASh neurons and LFPs to microinfusion of THC alone or in combination with either CYP or nor-BNI were determined by comparing neuronal frequency rates and local field potentials (LFP) oscillatory patterns between the 10-minute pre- vs. post-infusion recording epochs. A cell was considered to have changed its firing rate if there was a minimum of 20% difference in frequency rate from baseline. The electrode was used to simultaneously record LFPs. For histological analysis of extracellular NASh neuronal recording sites, recording electrode positions were marked with iontophoretic deposit of Pontamine Sky Blue dye (−20 μA, continuous current for 12–15 minutes). Brains were removed and post-fixed 24 h before being placed in a 25% formalin-sucrose solution for one week before sectioning (60 μm). Following this, sections were stained with neutral red and infusion/neuronal recording sites were confirmed with light microscopy.

### Experimental design and statistical analysis

ANOVA tests were performed using IBM SPSS Statistics software followed by LSD *post-hoc* testing. Sample sizes were pre-selected based on previous work. During electrophysiology experiments, an average of 5 cells were recorded per animals but some were excluded due to not meeting the cut-off criteria for MSNs.

## Results

### Histological analyses

**Figure 1:**
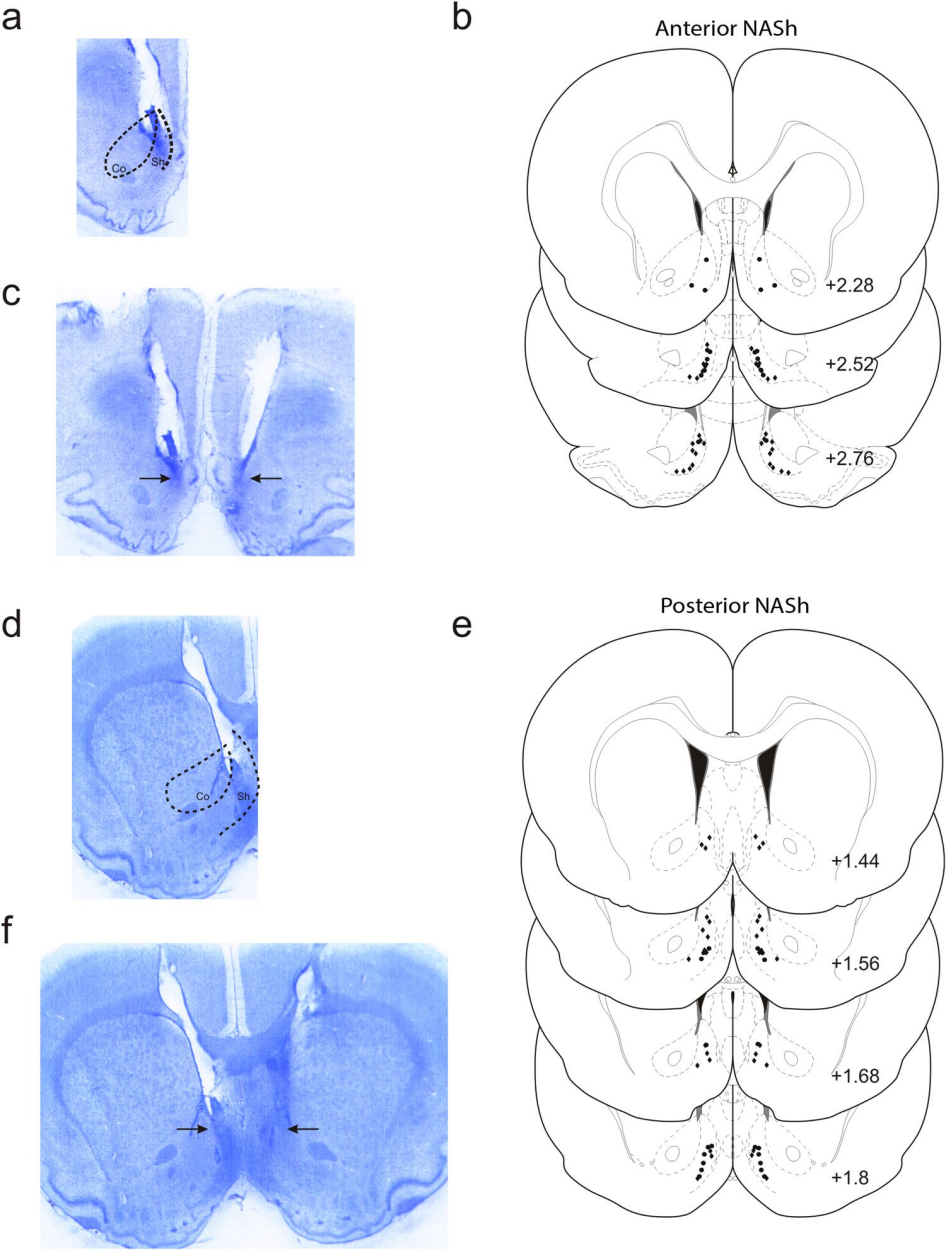
Histological analysis of intra-NASh microinjection sites. (**a**) Microphotograph of representative injector placement within the anterior portion of the nucleus accumbens shell. (**b**) Schematic representation of select intra-anterior-NASh injector locations; ● = 100 ng THC group, ◆ = 100 ng THC + 1 µg CYP. (**c**) Microphotograph of representative intra-anterior-NASh bilateral cannulae placements. (**d**) Microphotograph of representative injector placement within the posterior portion of the nucleus accumbens shell. (**e**) Schematic representation of select intra-posterior-NASh injector locations; ● = 100 ng THC group, ◆ = 100 ng THC + 1 µg CYP. (**f**) Microphotograph of representative intra-posterior-NASh bilateral cannulae placements.

Histological analysis revealed injector placements localized within the anatomical boundaries of the shell subdivision of the NASh, localized to the anterior vs. posterior anatomical divisions (see methods). In Fig. 1a, a representative microphotograph showing a typical intra-aNASh injector tip location is shown. In Fig. 1b, a representative microphotograph showing bilateral intra-aNASh injector locations is shown. In Fig. 1c, a schematic summary showing representative aNASh experimental group bilateral infusion locations is presented. In Fig. 1d, a representative microphotograph showing a typical intra-pNASh injector tip location is shown. In Fig. 1e, a representative microphotograph showing bilateral intra-pNASh injector locations is shown. In Fig. 1f, a schematic summary showing representative pNASh experimental group bilateral infusion locations is presented.

### Intra-NASh THC produces dose-dependent, anatomically dissociable reward or aversion effects via separate opioid receptor substrates

**Figure 2:**
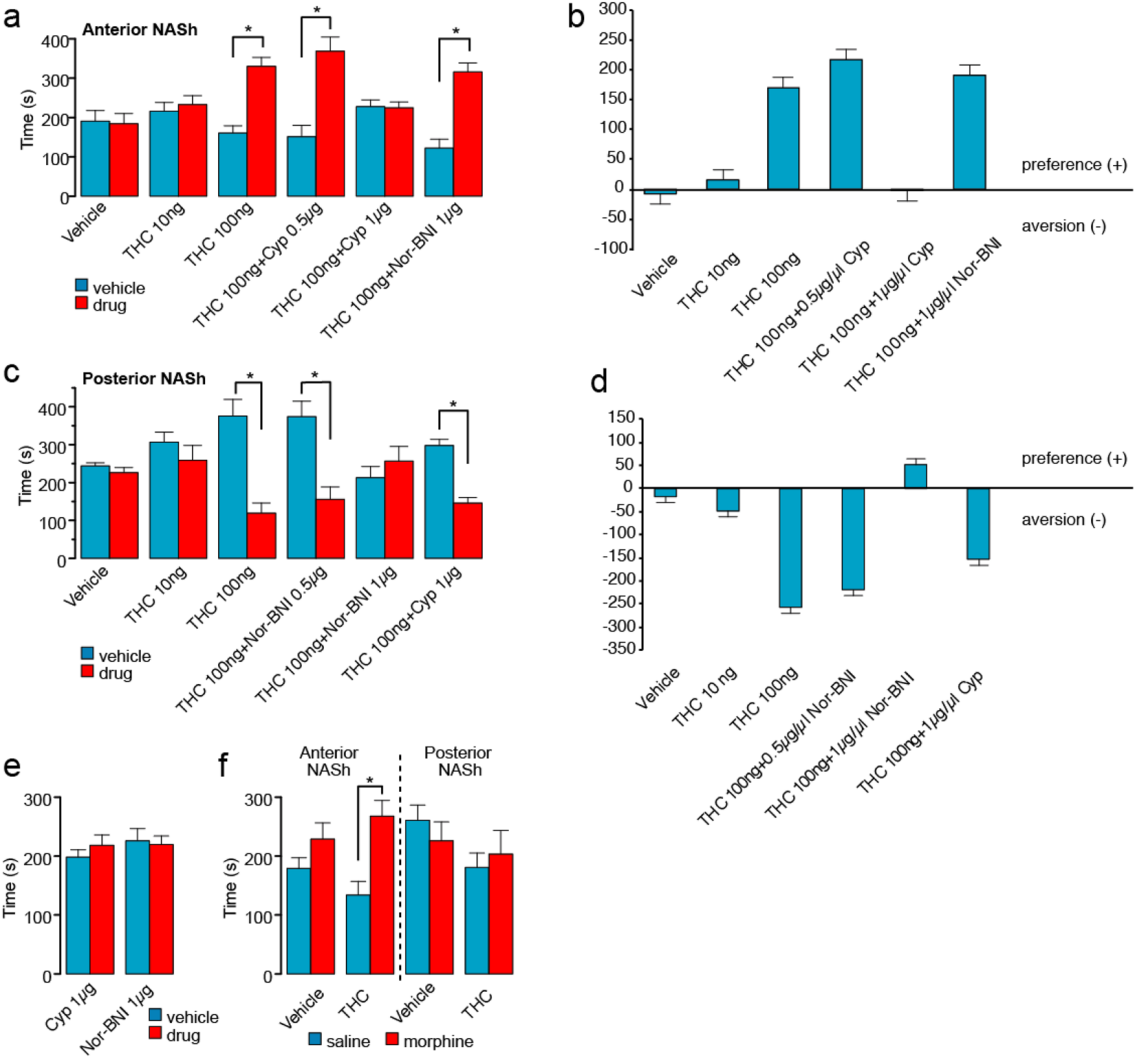
Effects of intra-NASh THC and selective MOR antagonists on place conditioning behaviors. (**a**) Anterior NASh (+2.5 mm from bregma) microinfusions of THC dose dependently increases preference for the drug paired side. Co-administration of cyprodime, but not Nor-BNI, dose-dependently blocks this effect. (**b**) Difference scores of the data presented in (**a**) showing the effect of THC in the anterior NASh. (**c**) In contrast, posterior NASh (+1.5 mm from bregma) THC dose dependently produces a conditioned place aversion. Co-administration of Nor-BNI, but not cyprodime, dose-dependently blocks this effect. (**d**) Difference scores of the data presented in (**c**) showing the effect of THC in the posterior NASh. (**e**) Infusions of the effective doses of cyprodime (1 μg) or Nor-BNI (1 μg) vs. VEH produce neither reward nor aversion effects in the CPP paradigm. (**f**) THC in the anterior (but not posterior) NASh (100 ng) selectively potentiates the rewarding effects of a sub-reward threshold CPP conditioning dose of morphine.

Given previous evidence demonstrating functional differences in anterior vs. posterior NASh region in reward vs. aversion processing^29^ and evidence demonstrating that cannabinoid signaling can modulate reward or aversion signals via MOR vs KOR receptor substrates^22^, we hypothesized that aNASh THC would produce rewarding effects through a MOR-dependent mechanism, whereas pNASh THC would produce aversive behavioral effects through a KOR-dependent. We first examined the effects of THC (10 ng or 100 ng/0.5 µl), directly in the anterior NASh, or in combination with the selective MOR antagonist [CYP (0.5 µg–1 µg/0.5 µl)] or KOR [*nor*-BNI (1 µg/0.5 µl)] antagonist using an unbiased CPP procedure (*see methods*). Two-way ANOVA comparing times spent in the THC-paired vs. VEH- paired environments revealed a main effect of environment (*F*_(1,110)_ = 46.094, *p* < 0.001) and a significant treatment x environment interaction (*F*_(5,110)_ = 9.802, *p* < 0.001; Fig. 2a). *Post-hoc* analyses showed that intra-aNASh THC produced dose-dependent CPP for THC-paired environments as rats receiving a higher THC dose showed significant CPP (100 ng; n = 10; *p* = 0.002) vs. rats receiving the lower dose (10 ng; *n* = 10; p > 0.05; Fig. 2a). Co-administration with the selective MOR antagonist, cyprodime, dose-dependently blocked the rewarding effects of aNASh THC as rats receiving a lower dose of CYP (0.5 μg) + THC (100 ng) still showed significant CPP for THC-paired environments (0.5 μg; n = 10; *p* = 0.01) while a higher dose of CYP (1 μg; *n* = 11) blocked THC CPP (p > 0.05; Fig. 2a). In contrast, rats receiving THC co-administration with a selective KOR antagonist, *nor*-BNI, still displayed robust CPP for THC-paired environments (1 μg; n = 10; *p* = 0.002). VEH control rats (*n* = 10) displayed neither preference nor aversion for either environment (*p* > 0.05; Fig. 2a). The same data are presented in the form of difference scores to highlight the relative change in time spent in each environment (Fig. 2b). No significant difference was observed for locomotor activity between groups when comparing activity in the different drug vs. VEH conditioning environments (data not shown). Thus, THC in the anterior NASh produces dose-dependent rewarding effects which are dependent upon local MOR transmission but independent of KOR transmission.

Next, we examined the potential motivational effects of THC in the posterior NASh. Again, using two different doses of THC (10 ng and 100 ng/0.5 µl), two doses of the selective KOR antagonist, *nor*-BNI (0.5 µg and 1 µg/0.5 µl) in combination with the higher dose of THC, *nor*-BNI alone (1 µg/0.5 µl) or the higher dose of THC in combination with the MOR antagonist, CYP (1 µg/0.5 µl). Two-way ANOVA comparing times spent in THC vs. VEH-paired environments revealed a significant main effect of environment (*F*_(1,116)_ = 26.584, *p* < 0.001) and a significant treatment x environment interaction (*F*_(5,116)_ = 7.950, *p* < 0.001; Fig. 2c). *Post-hoc* analyses showed that intra-pNASh THC produced dose-dependent conditioned place aversions (CPA) for THC-paired environments as rats receiving a higher THC dose showed significant CPA (100 ng; n = 11; p = 0.004) vs. rats receiving the lower THC dose, who showed neither preference nor aversion behaviors (10 ng; n = 10; p > 0.05; Fig. 2c). THC co-administration with the selective KOR antagonist, *nor*-BNI, dose-dependently blocked the aversive effects of pNASh THC as rats receiving a lower dose of *nor*-BNI (0.5 μg) + THC (100 ng) still showed significant CPA for THC-paired environments (0.5 μg; *n* = 11; *p* = 0.009) while the higher dose of *nor*-BNI blocked THC CPA(1 μg; *n* = 11; p > 0.05; Fig. 2c). In contrast, rats receiving THC co-administration with the selective MOR antagonist, cyprodime, still displayed robust CPA for THC-paired environments (1 μg; n = 11; *p* < 0.001). Rats receiving vehicle (*n* = 10) displayed neither preference nor aversion for either environment. The same data are presented in the form of difference scores to highlight the relative change in time spent in each environment (Fig. 2d). No significant difference was observed for locomotor activity between groups. Thus, THC in the posterior NASh produces dose-dependent aversion effects which are dependent upon local KOR transmission but independent of MOR transmission.

Finally, to control for the potential motivational effects of either *nor*-BNI or CYP in and of themselves, separate control groups received either the effective dose of intra-aNASh CYP (1 µg; n = 9) vs. VEH or intra-pNASh *nor*-BNI (1 μg; n = 10) vs. VEH. Neither group displayed a preference or aversion for drug vs. VEH-paired environments (p’s > 0.05; Fig. 2e). Together, this data demonstrates a double-dissociation between the rewarding and aversive motivational effects of THC in the anterior vs. posterior NASh, mediated through separate MOR vs. KOR signaling mechanisms, while blockade of aNASh MOR or pNASh KOR transmission has no motivational effects in and of itself.

### Intra-aNASh THC potentiates sub-threshold morphine reward salience

Given our findings that aNASh THC produced robust rewarding effects through a MOR-dependent substrate and previous evidence showing that stimulation of µORs within the NASh can potentiate drug reward salience^30^, we next examined how intra-NASh THC may modulate the motivational effects of an exogenous opioid, morphine, using a sub-reward threshold conditioning dose of morphine (0.05 mg/kg; i.p.; *see methods*). To control for the previously characterized rewarding or aversive effects of THC (in the anterior vs. posterior NAc, respectively; Fig. 2a,c), 4 separate groups received either intra-aNASh or pNASh THC (100 ng) prior to both morphine and saline conditioning sessions or aNASh or pNASh VEH. Thus, the motivational properties of THC were counterbalanced across both saline and morphine conditioning environments, meaning that place preferences/aversions would be associated with the potential effects of morphine vs. saline, rather than the effects of intra-NASh THC in and of itself. Two-way ANOVA comparing times spent in morphine vs. saline-paired environments revealed a significant main effect of treatment on times spent in morphine vs. saline-paired environments (*F*_(3,24)_ = 3.702, *p* = 0.026). *Post-hoc* analyses revealed that there was no significant difference between intra-pNASh THC (100 ng; n = 7) vs. vehicle CPP times, as both groups displayed neither preference nor aversions to either environment (n = 7; *p’s* > 0.05; Fig. 2e). In contrast, rats receiving intra-aNASh THC (100 ng; n = 7), displayed a significant CPP for morphine-paired environments relative to VEH controls (n = 7; *p* = 0.030; Fig. 2e). Thus, consistent with the ability of aNASh THC to produce rewarding effects through a MOR-dependent substrate, aNASh THC potentiated the reward salience of normally sub-reward threshold morphine conditioning cues. In contrast, THC in the pNASh had no effect on sub-threshold morphine CPP behaviors.

### Intra-NASh THC has no effect on sucrose reward processing

**Figure 3:**
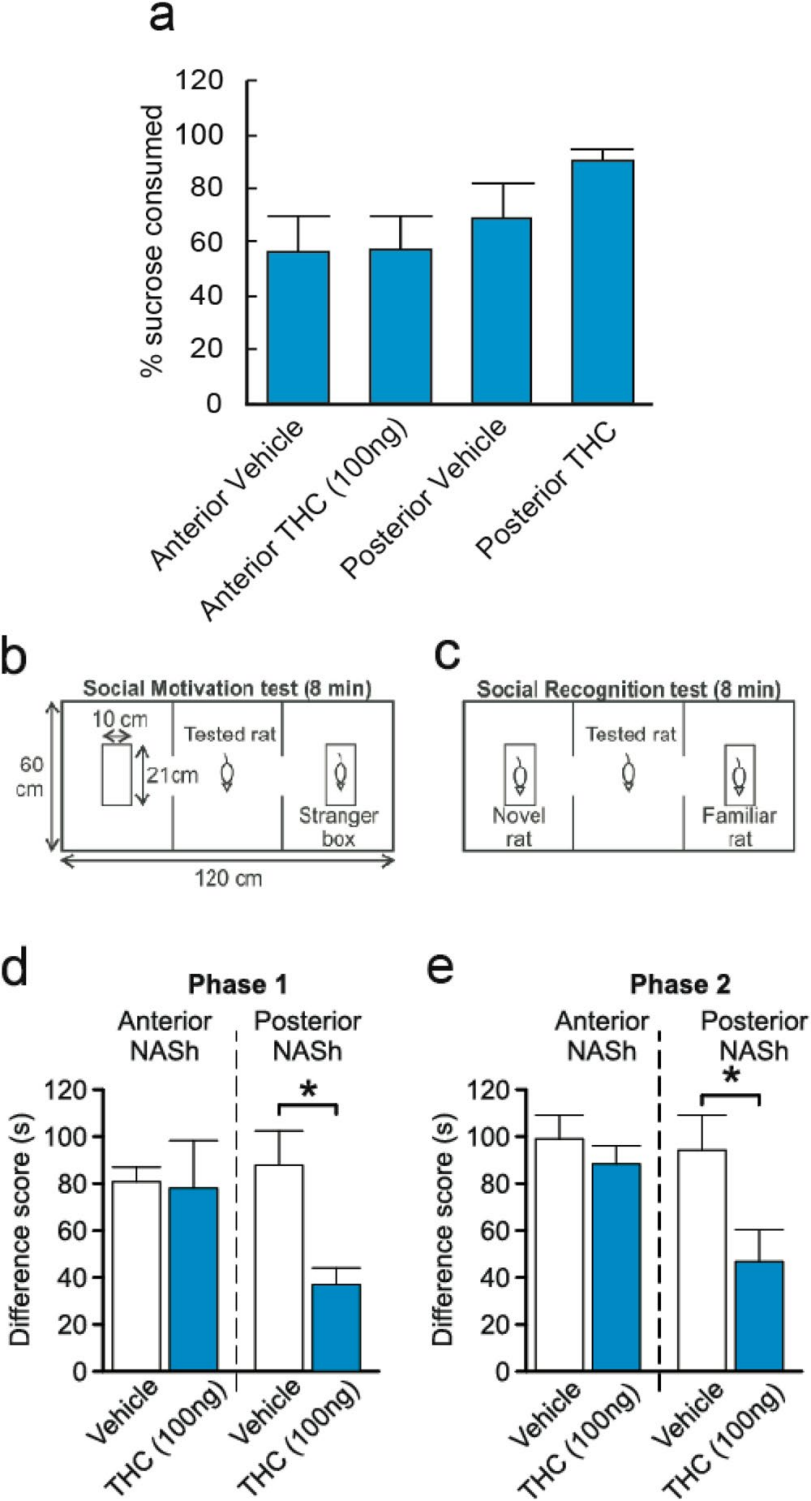
Effects of intra-NASh THC on sucrose preference and social recognition. (**a**) THC has no significant effect on the percent consumption of sucrose vs total liquid consumed. (**b**,**c**) Summary of apparatus and experimental procedure for the sociability and social recognition test phases. (**d**) Microinfusions of THC in the pNASh, but not the aNASh, significantly reduced sociability scores *p < 0.05. (**e**) Microinfusions of THC in the pNASh, but not the aNASh, significantly reduced social recognition scores *p < 0.05.

To determine if the effects of intra-NASh THC on affective processing may modulate non-drug-related motivational effects, we next examined the processing of natural, sucrose-related reward (*see methods*). Percent sucrose consumed was calculated by dividing the amount of sucrose consumed by the amount of total liquid (water plus sucrose) consumed. Although there was a trend towards an increase in sucrose consumption by rats receiving intra-pNASh THC, statistical comparison showed no significant difference between rats receiving intra-aNASh vehicle (*n* = 7) and intra-aNASh THC (*n* = 8; *t*_(13)_ = −0.051, *p* > 0.05) or intra-pNASh vehicle (*n* = 8) and intra-pNASh THC (*n* = 8, *t*_(14)_ = 0.152, *p* > 0.05; Fig. 3a), indicating that while THC in the aNASh is capable of potentiating drug-related reward salience (morphine), this effect does not influence a natural reward cue (sucrose).

### THC induces social interaction and cognition deficits selectively in the posterior NASh

Previous studies have demonstrated that cannabinoid signaling can strongly modulate social behavioral phenomena through actions in the NASh^29^. Social behaviors are also naturally rewarding for rats therefore social interaction can also act as another model of natural reward. Therefore, we examined the potential effects of intra-NASh THC on social motivation behaviors and cognition (social memory). A simplified diagram of the experimental procedure is presented in Fig. 3b,c. In phase 1, sociability scores (measuring motivation to interact with a novel rat) were calculated by measuring times spent interacting with a novel rat and subtracting times spent interacting with an empty box. ANOVA showed a main effect of treatment on Phase 1 sociability scores (*F*_(3,26)_ = 3.156, *p* = 0.042). *Post-hoc* analyses revealed a significant difference between intra-pNASh THC (*n* = 8) and intra-pNASh vehicle (*n* = 8; *p* = 0.009), intra-aNASh vehicle (*n* = 7; *p* = 0.029) and intra-aNASh THC (*n* = 7; *p* = 0.038) (Fig. 3d). No significant differences were observed between any other groups. In phase 2, social memory scores were calculated by taking times spent with a new, novel rat and subtracting times spent with the previously encountered, familiar rat. ANOVA showed a main effect of treatment for social memory scores (*F*_(3,26)_ = 3.516, *p* = 0.029). *Post-hoc* testing revealed a significant difference between intra-pNASh THC (*n* = 8) and intra-pNASh vehicle (*n* = 8; *p* = 0.013), intra-aNASh vehicle (*n* = 7; *p* = 0.009) and intra-aNASh THC (*n* = 7; *p* = 0.013) (Fig. 3e). Thus, intra-NASh THC selectively impairs natural social motivation and social memory cognition selectively in the posterior region of the NASh.

### THC inhibits spontaneous medium spiny neuron activity in the anterior NASh through a MOR-dependent mechanism

**Figure 4:**
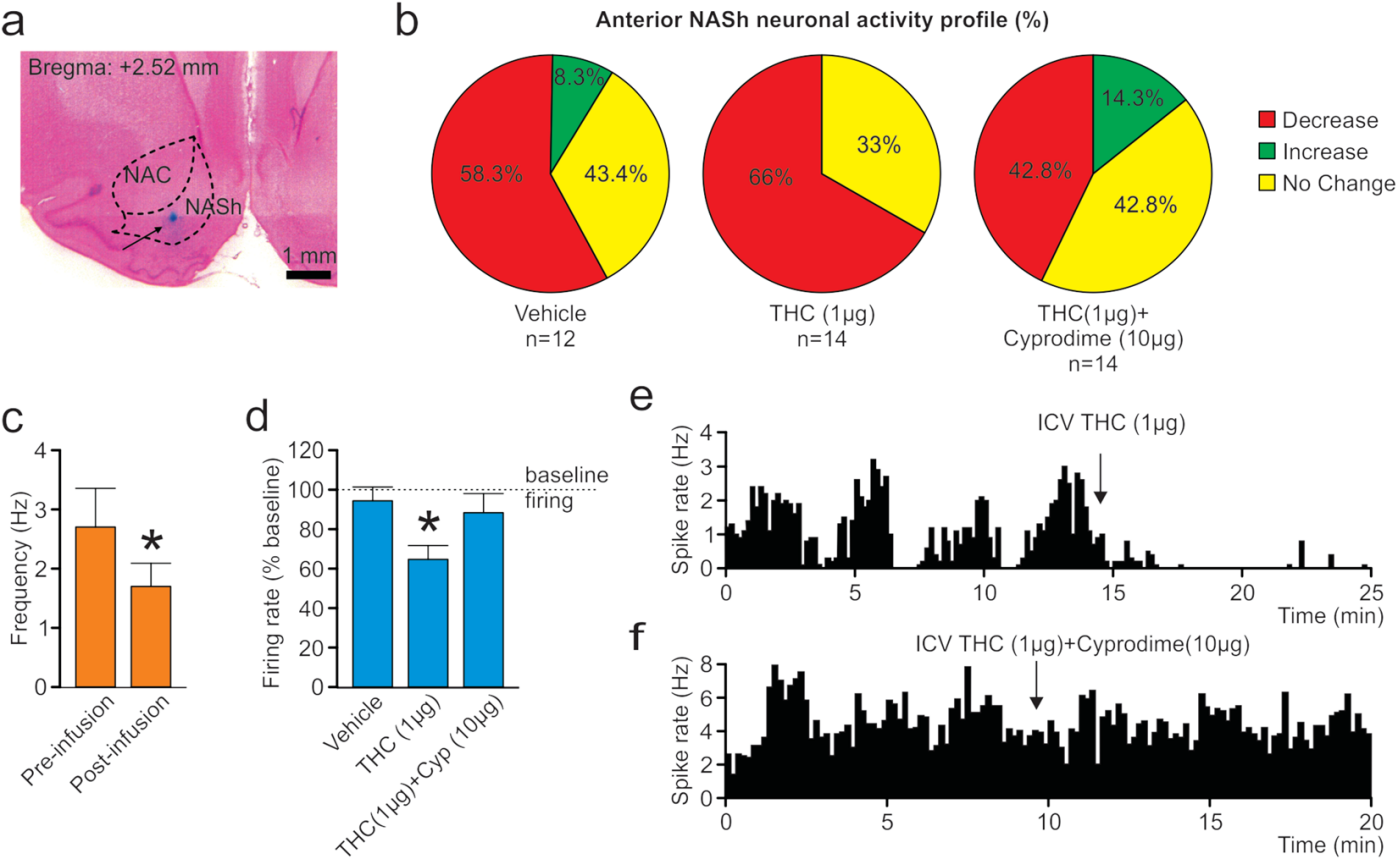
Effects of ICV THC and CYP on anterior NASh medium spiny neurons activity patterns. (**a**) Representative microphotograph showing typical intra-aNASh *in vivo* MSN recording location (**b**) Summary of experimental neuronal groups showing relative changes (no change, increase, or decrease) in firing frequencies following ICV pharmacological treatments (**C**) ICV THC significantly decreased spontaneous aNASh MSN neuronal firing frequency. (**d**) ICV THC alone (1 µg/μl) caused a significant decrease in spontaneous aNASh MSN neuronal firing frequency rates vs baseline. This inhibitory effect was reversed by co-administration of the behaviorally effective dose of CYP (10 µg/μl). (**e**) Sample rastergram showing typical aNASh MSN response pattern following ICV THC (1 µg/µl) infusion (arrows indicate intra-NAc infusion event). (**f**) Sample rastergram showing typical aNASh MSN response pattern following ICV THC (1 µg/µl) and CYP (10 µg/µl) infusion, demonstrating the block of THC’s neuronal effects with MOR blockade (arrows indicate intra-NAc infusion event).

The activity states of NASh MSN neurons are strongly correlated with reward vs. aversive motivational states^17^ and we have previously demonstrated that cannabinoid CB1 transmission can produce rewarding effects by inhibiting NASh MSN neurons or aversive effects by activating these same neurons^18^. To determine if the anatomically localized effects of THC on reward or aversion were correlated with MSN activity state modulation, we next performed *in vivo* single-unit recordings in the posterior and anterior NASh, combined with ICV infusions of THC. We used a THC dose 10x our highest behaviorally effective dose (1 µg/µl) for these systemic electrophysiological recording studies to control for potential CSF diffusion effects (*see methods*). First, a total of n = 15 MSNs were isolated in the aNASh and we compared frequency rates pre vs. post THC administration (see Fig. 4a for representative aNASh recording location). Population analysis of aNASh MSNs revealed that 66.6% showed decreased activity, 0% increased, and 33.3% were unchanged, relative to baseline, following ICV THC administration (Fig. 4b). Thus, a plurality of aNASh MSNs showed a decrease in spontaneous firing frequency following ICV THC administration. An analysis of average firing frequency recorded 10 min pre vs. post ICV THC infusion revealed that THC significantly decreased firing rates in the aNASh (*t*_(14)_ = 2.738, *p* = 0.016; Fig. 4c).

Given our previous behavioral findings (Fig. 2a,c) showing THC-induced reward or aversion mediation through differential MOR vs. KOR transmission, respectively, we next performed *in vivo* single-unit recordings in the posterior and anterior NASh to determine if the anatomically dissociable effects of THC on MSN activity states may similarly depend upon differential opioid receptor signaling. Therefore, we performed co-administration studies using THC + CYP, or THC + *nor*-BNI, using 10x our behaviorally effective doses of THC (1 µg/µl), CYP (10 µg/µl), and *nor*-BNI (10 µg/µl).

In the aNASh, we sampled a total of n = 40 MSNs (Vehicle: n = 12, THC: n = 14, THC + CYP: n = 14). For rats receiving ICV vehicle, 58.3% of neurons showed no change, 8.3% increased and 43.4% showed decreased activity. For rats receiving ICV THC + CYP, 42.8% of neurons showed no change, 14.3% increased and 42.8% showed decreased activity (Fig. 4b, far right side). Analyses of pre vs. post infusion activity rates for aNASh MSNs revealed average changes from baseline of −4.9% for rats treated with vehicle, −34.5% for rats treated with THC, and −11% with THC + CYP. ANOVA comparing groups revealed a significant main effect of treatment (*F*_(2,38)_ = 3.889, *p* = 0.029; Fig. 4d). *Post-hoc* analysis revealed that rats treated with ICV THC showed significantly decreased activity relative to VEH controls (*p* = 0.014; Fig. 4d) and from the THC + CYP group (*p* = 0.039; Fig. 4d). The vehicle group did not differ significantly from the THC + CYP group (*p* > 0.05; Fig. 4d). Co-treatment with CYP, therefore, reversed THC-induced inhibition of spontaneous MSN activity. A representative rastergram showing a typical inhibitory response to THC in the aNASh is shown in Fig. 4e. A sample neuronal rastergram from a THC + CYP treated rat are shown in Fig. 4f, showing the typical blockade of THC-induced neuronal inhibition.

### THC increases spontaneous medium spiny neuronal activity in the posterior NASh through a KOR-dependent mechanism

**Figure 5:**
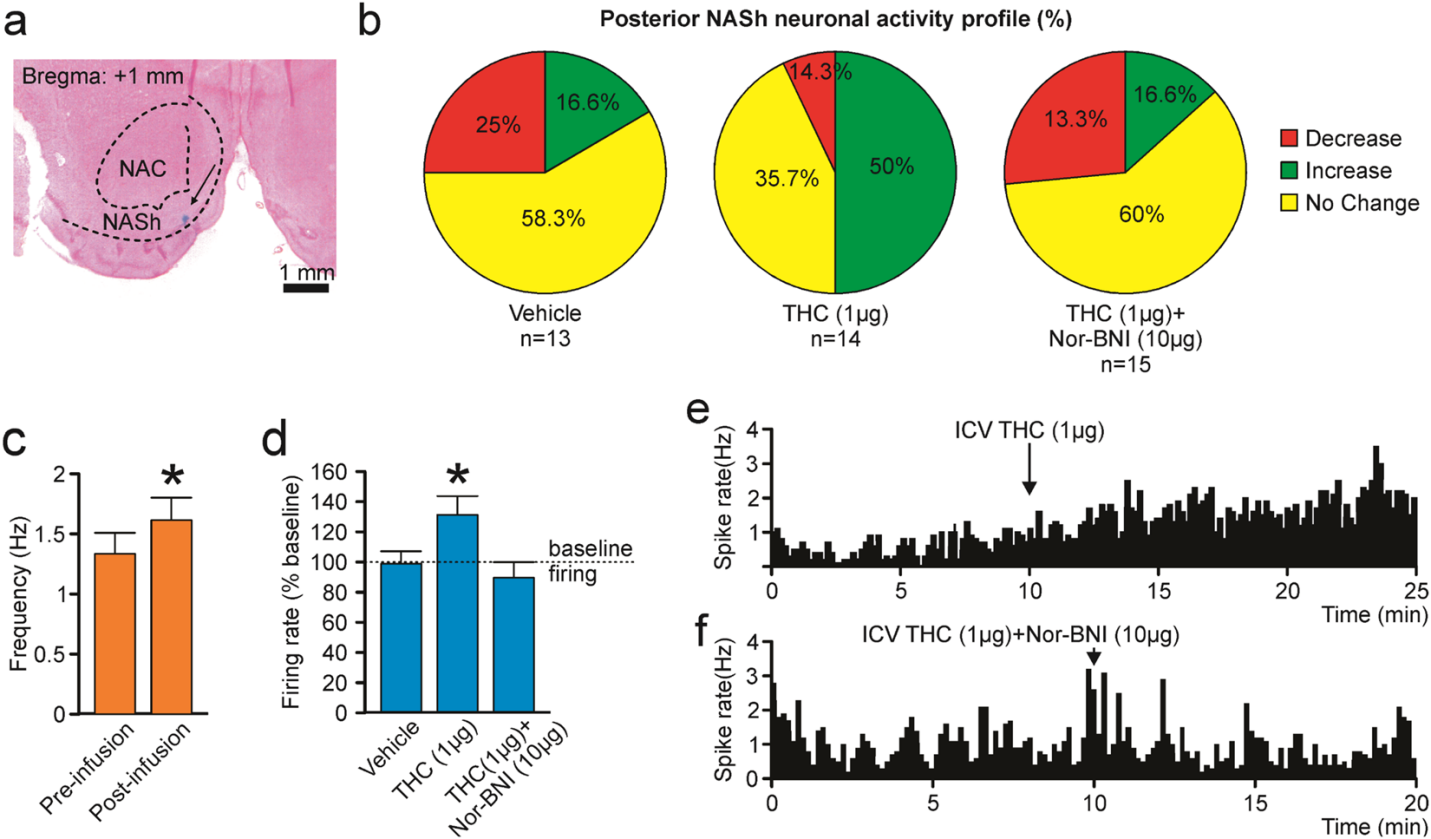
Effects of ICV THC and nor-BNI on MSN activity patterns in the pNASh. (**a**) Representative microphotograph showing typical intra-pNASh *in vivo* MSN recording location. (**b**) Summary of experimental neuronal groups showing relative changes (no change, increase, or decrease) in firing frequencies following ICV pharmacological treatments. (**c**) ICV THC significantly increased spontaneous pNASh MSN neuronal firing frequency. (**d**) ICV THC alone (1 µg/μl) caused a significant increase in spontaneous aNASh MSN neuronal firing frequency rates vs baseline activity. This excitatory effect was reversed by co-administration of the behaviorally effective dose of nor-BNI (10 µg/μl). (**e**) Sample rastergram showing typical pNASh MSN response pattern following ICV THC (1 µg/µl) infusion (arrows indicate intra-NAc infusion event). (**f**) Sample rastergram showing typical aNASh MSN response pattern following ICV THC (1 µg/µl) and nor-BNI (10 µg/µl) infusion (arrows indicate intra-NAc infusion event).

For MSN neurons recorded in the pNASh (n = 14; see Fig. 5a for representative pNASh recording location), population analysis revealed that 14.3% of MSNs showed decreased activity, 50% increased and 35.7% showed no change, relative to baseline, following ICV THC administration (Fig. 5b). Thus, a plurality of pNASh MSN neurons show an inhibitory response to ICV THC administration. Analysis of average firing frequencies recorded 10 min pre vs. post ICV THC revealed that THC significantly increased firing rates relative to baseline (*t*_(13)_ = −2.288, *p* = 0.04; Fig. 5c). Given our previous findings showing that the effects of pNASh THC were dependent upon a KOR-transmission substrate, we next sampled a total of n = 42 pNASh MSNs (Vehicle: n = 13, THC: n = 14, THC + *nor*-BNI: n = 15). Population analyses revealed that for rats receiving ICV vehicle, 58.3% of neurons showed no change, 16.6% increased and 25% showed decreased activity. For rats receiving ICV THC + *nor*-BNI, 60% of neurons showed no change, 13.3% increased and 26.6% showed decreased activity (Fig. 5b, far right side). Analyses of pre vs. post infusion activity rates for pNASh MSNs revealed average changes from baseline of −1.7% for rats treated with vehicle, +31.3% for rats treated with THC, and −9.3% with THC + CYP. ANOVA comparing groups revealed a significant main effect of treatment (*F*_(2,38)_ = 4.085, *p* = 0.025; Fig. 5d). *Post-hoc* analysis revealed that rats treated with ICV THC showed significantly decreased activity relative to VEH controls (*p* = 0.043; Fig. 5d) and from the THC + *nor*-BNI group (*p* = 0.01; Fig. 5d). The vehicle group did not differ significantly from the THC + *nor*-BNI group (*p* > 0.05; Fig. 5d). Thus, co-treatment with *nor*-BNI reversed THC-induced increases in spontaneous MSN activity in the posterior NASh. A representative rastergram showing a typical excitatory response pattern to THC administration in the pNASh is shown in Fig. 5e. A representative neuronal rastergram from a THC + *nor*-BNI treated rat is shown in Fig. 5f, showing a typical blockade of THC-induced neuronal excitation following in the presence of the KOR antagonist.

### THC produces differential changes in the power of high-frequency γ-oscillations

**Figure 6:**
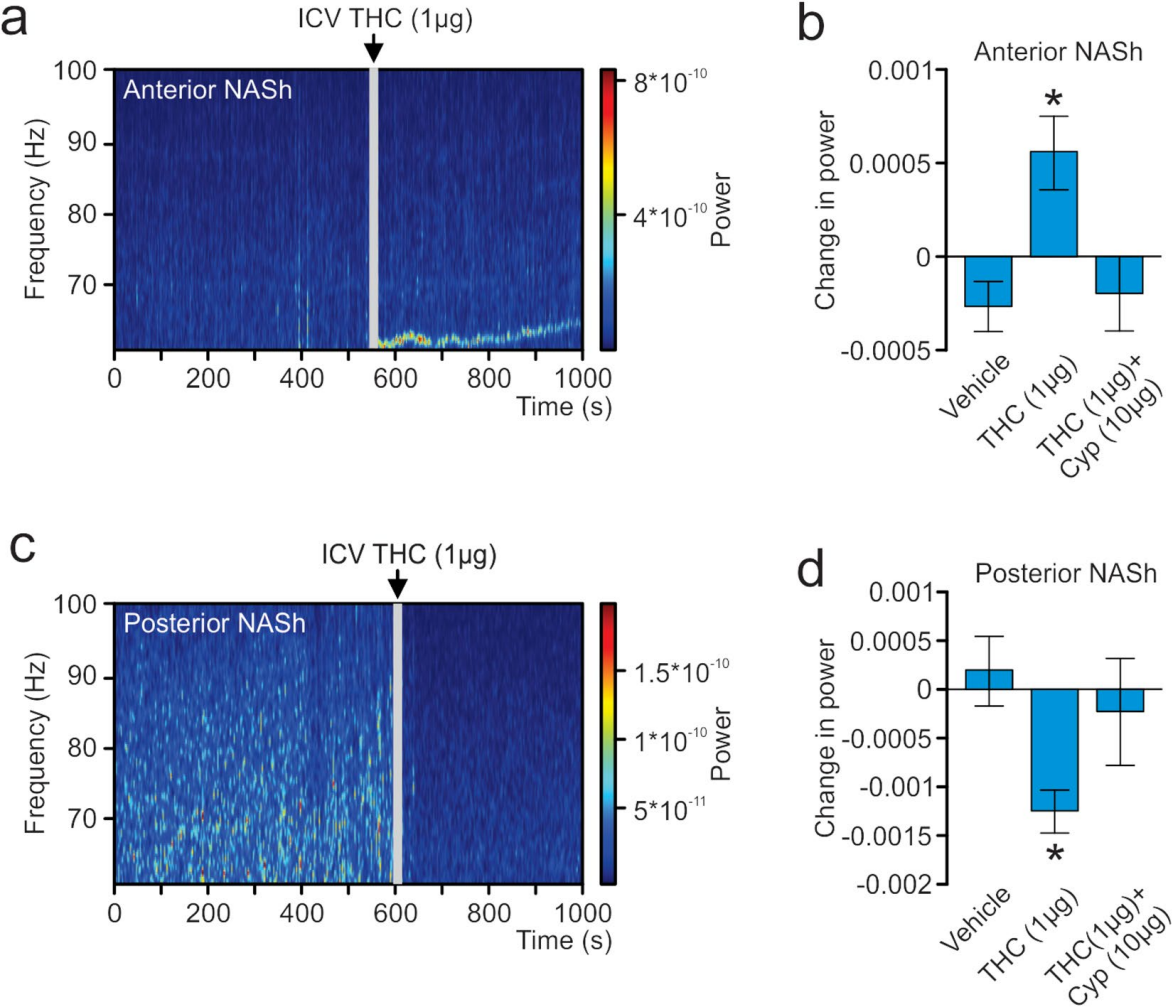
Effect of ICV THC on the power of high-frequency gamma oscillations in the local field potential signal in the NASh. (**a**) Sample spectrograph showing typical high-frequency γ-oscillations in the aNASh following ICV infusion of THC (**b**) ICV THC significantly decreases the power of high-frequency γ-oscillations in the aNASh. *p < 0.05 from the other two groups. (**c**) Sample spectrograph showing typical high-frequency γ-oscillations in the pNASh following ICV infusion of THC (**d**) ICV THC significantly increases the power of high-frequency γ-oscillations in the pNASh.

In the above described studies, LFPs were recorded concurrently with single-unit activity. The signal was divided into bins of 2 seconds and 410 different frequency values. An analysis was performed to determine the power each frequency had on the signal. A sample spectrograph of an aNASh LFP recording is shown in Fig. 6a. ANOVA comparing the power of high-frequency γ-oscillations between treatment groups revealed a significant main effect of treatment (*F*_(2,35)_ = 3.963, *p* = 0.028; Fig. 6b). *Post-hoc* analyses revealed that rats treated with ICV THC showed significantly increased power of high-frequency γ-oscillations relative to VEH controls (*p* = 0.010; Fig. 6b) or rats treated with ICV THC + CYP (*p* = 0.045; Fig. 6b). The VEH group also did not differ significantly from the ICV THC + CYP group (*p* > 0.05; Fig. 6b). THC, therefore, increased the power of high-frequency γ-oscillations in the aNASh and this effect was reversed by co-treatment with a MOR antagonist.

Next, we ran an analysis on pNASh LFPs. A sample spectrograph of a pNASh LFP is shown in Fig. 6c. ANOVA revealed a main effect of treatment (*F*_(2,38)_ = 5.130, *p* = 0.011; Fig. 6d). *Post-hoc* analyses revealed that THC treatment significantly decreased the power of high-frequency γ-oscillations relative to VEH controls (*p* = 0.004; Fig. 6Dd) or rats treated with ICV THC + *nor*-BNI (*p* = 0.019; Fig. 6c). Rats treated with ICV vehicle did not differ significantly from rats treated with ICV THC + *nor*-BNI (*p* > 0.05; Fig. 6c). Thus, in direct contrast to the anterior NASh, pNASh THC decreased the power of high-frequency γ-oscillations, through a KOR-dependent signaling mechanism.

## Discussion

The nucleus accumbens serves as a critical nexus point for the integration of affective information from numerous inputs, including the ventral tegmental area (VTA), amygdala, ventral hippocampus (vHIPP) and prefrontal cortex (PFC). In addition, THC has been shown to strongly modulate intra-NAc signaling from these regions, including DAergic signals from the VTA^31–34^ and glutamatergic signals from the PFC and vHIPP^26,35–37^. Previously, we and others have reported that THC directly within the NAc can strongly modulate mesolimbic DAergic activity and NAc neuronal activity states^5,31,33,34^. Furthermore, THC has been shown to directly activate VTA DA neurons^6^. Nevertheless, the precise neuroanatomical, neuronal and pharmacological mechanisms by which THC produces localized, bivalent, rewarding or aversive motivational effects within the NAc has not previously been characterized.

In the present study, we demonstrate not only that THC produces bivalent effects on reward and aversion processing, but identify anatomically, pharmacologically and neuronally dissociable mechanisms, directly in the in the anterior vs. posterior NASh that are responsible for these effects. We report a functional double-dissociation of the effects of THC such that intra-NASh THC infusions produced robust µOR-dependent reward effects selectively in the aNASh, but κOR-dependent aversive behavioural effects in pNASh. In addition, intra-aNASh THC selectively potentiated morphine-related reward salience but not natural, sucrose reward salience. In contrast, intra-pNASh THC reduced social motivation and social memory processing, without influencing morphine-related reward processing. Finally, we found that ICV infusions of THC produced a predominant decrease in MSN activity, consistent with an accumbal neuronal reward signature^17,25^. In direct contrast, THC in the pNASh induced a significant increase in MSN neuronal activity with a concomitant decrease in high-frequency γ-oscillations, consistent with aversion-related neuronal activity within the NAc^17,25^. Together, these findings identify critical functional differences in the NASh related to THC modulation of affective processing and demonstrate that distinct OR substrates are responsible for these seemingly contradictory effects.

The functional differences in OR signaling between the aNASh and the pNASh are not fully understood. However, previous studies have suggested differential effects of these OR subtypes on DA-mediated phenomena. For example, NASh activation of MORs stimulates DA release while KOR activation decreases DA release^38^. Since DA transmission in the NASh is strongly linked to both motivational processing^39^ and addiction behaviors^34,40^, differential changes in DA release caused by stimulation of distinct NAc OR substrates could potentially underlie these bivalent affective phenomena. Previous studies have demonstrated the ability of cannabinoids to regulate mesolimbic DA release^33,41,42^ and enhanced phasic DA release induced by psychoactive drugs is regulated by local cannabinoid receptor signaling^43^.

Clinical and pre-clinical studies have demonstrated biphasic effects of THC on reward and aversion processing^1–3^. For example, strain-dependent differences in sensitivity to the rewarding or aversive stimulus properties of THC has been demonstrated in rodents (Parker and Gillies, 1995). In humans, THC exposure produces differential rewarding or aversive effects via modulation of striatal activity states and can disrupt emotional processing^44–47^. Like many drugs of abuse, THC elicits striatal DA release^44^ and can reduce limbic network activity when processing negative emotional stimuli^45^. THC, however, also causes paranoia, anxiety and negative mood^46,47^ which increases with dose^48^. The present findings suggest that relative sensitivity to the motivational properties of THC may depend upon distinct effects of THC within anterior vs. posterior NAc regions via dissociable OR signaling mechanisms. These data are in contrast with a previous report showing that infusions of THC into the pNASh caused a mild conditioned place preference^49^. The CPP procedure used in this study, however, had several methodological differences, including fewer conditioning days and longer place conditioning sessions^49^. However, the present results are consistent with previous infusion studies performed in the pNASH using the endocannabinoid, anadamide^50^ which also reported aversive-like behavioural effects. Regardless, future studies are required to determine if similar regional differences in the human ventral striatum may similarly underlie the bivalent affective properties of THC.

We observed deficits in social motivation and recognition memory following THC infusions into the pNASh, but no effects in the aNASh. While social interaction is naturally rewarding for rodents, these results can potentially be interpreted as THC-induced modulation of the hedonic value of social cues, consistent with our observed aversive place conditioning effects induced by pNASh THC. We also observed deficits in social recognition memory following the first phase of the interaction test. This effect might indicate either a lack of motivation to spend time with the second novel subject in phase 2, or a lack of recall of the previously encountered subject in phase 1. However, given that the THC rats (pNASh) in Phase 2 did not decrease overall interaction times but rather demonstrated a leveling off of interaction between the novel and familiar test subjects, this effect is suggestive of an impairment in recognition memory, rather than the production of an aversive associative state linked to the subject rats.

The cannabinoid and opioid receptor systems functionally interact during the processing of motivational behaviors^16,22,51^. For example, systemic THC administration has been shown to increase heroin self-administration in rats^52^ and intra-NAc CB_1_ receptor blockade has been shown to attenuate morphine self-administration^53^. Consistent with these reports, the present study identifies the anterior NASh region as a local accumbal zone critical for modulating opioid-related reward salience. Beyond the NAc, we have previously reported that intra-vHIPP CB1R activation strongly increases the reward salience of morphine^26^. In addition, CB1R activation in the PFC was shown to switch the hedonic valence of systemic or intra-VTA morphine from rewarding to aversive, through dissociable μOR vs. κOR-dependent receptor mechanisms, directly in the VTA^22^. Interestingly, intra-basolateral amygdala (BLA) activation or blockade of CB1R transmission has been shown to switch morphine reward behaviors into aversion effects and potentiate the reward salience of sub-threshold morphine, via functional modulation of MSN neuronal states in the NAc^18^. Thus, inhibition of CB1R signaling in the BLA with an antagonist of CB1R, was shown to potentiate the rewarding effects of morphine by causing inhibition of NASh MSN activity states. The present study reveals a novel, THC-mediated mechanism directly in the aNASh, that similarly potentiates opioid reward salience via inhibition of MSN neuronal activity. Thus, the relative activity levels of NAc MSN neuronal populations appears to be a critical mechanism regulating not only the motivational valence of THC itself, but also in the interaction of CB1R signaling with the processing of opioid-related reward or aversion behaviors.

Mesolimbic oscillatory states are closely linked to the functional activity of individual neurons during the processing of motivation, drug addiction and psychosis^54–56^. More specifically, γ-oscillations have been implicated in sensory integration, associative learning and cognition^54^. For example, previous studies have demonstrated that local firing activity of individual mesolimbic neurons is mediated by regionally distinct changes in γ-oscillations in the ventral striatum of rats during reward processing^19^ and there is increased striatal γ-oscillation activity immediately following associative reward delivery^57^. Such reward-related increases in accumbal γ-oscillatory states are consistent with the present study wherein we found that THC effects in the anterior NASh, which produced conditioned reward effects, potentiated the reward salience of normally non-salient opioid conditioning cues and strongly increased γ-oscillation levels. Indeed, the role of γ-oscillations in striatal reward processing is also demonstrated in humans during the processing of monetary rewards or losses, which are correlated with distinct γ-oscillation patterns in the NAc, suggesting that striatal γ-oscillation patterns may serve as a gating mechanism for the relative encoding of rewarding or aversive valences during motivated behaviors^55^. The present findings reveal for the first time that striatal γ-oscillation patterns are similarly linked to THC-dependent affective processing and suggest that THC-induced reward states are associated with potentiated γ-oscillations.

In contrast, THC produced aversive conditioned effects and social interaction and cognition deficits selectively in the posterior NASh. Interestingly, these effects coincided with the inhibition of γ-oscillatory levels selectively in the posterior NASh. Dysregulated striatal γ-oscillatory states have been linked to the negative symptoms of schizophrenia, which include anhedonic effects and social withdrawal and cognition deficits^58^. Thus, the present findings demonstrate for the first time, a potential link between THC-induced schizophrenia-like endophenotypes and disruptions in γ-oscillatory states in an anatomically localized region of the ventral striatum. Future studies are required to more fully explore the potential translational implications between these dissociable anterior vs. posterior oscillatory signatures, and THC-induced neuropsychiatric deficits.

Beyond signaling reward or aversion states, changes in γ-oscillation states have been linked to the psychotomimetic effects of THC. For example, THC exposure induces strong dysregulation in γ-oscillation states similar to those observed in schizophrenia^20,59–61^. Due to increasing evidence demonstrating functional links between THC exposure and long-term neuropsychiatric side-effects^62,63^, the present findings have important implications for how THC may lead to disturbances in emotional regulation via γ-oscillation alterations. In terms of drug-related effects, previous studies have demonstrated that specific patterns of γ-oscillations within the NAc were present during aversive opioid withdrawal states^64^, suggesting a link between opioid-related aversion signals and striatal γ-oscillation disturbances. Thus, altered mesolimbic γ-oscillation states may be critical biomarkers for striatal-dependent processing of rewarding or aversive motivational states and THC modulation of cannabinoid receptor signaling may serve to differentially control reward or aversion processing in distinct anterior vs. posterior regions of the NAc.

In summary, the results of the present study reveal several novel mechanisms to account for how THC differentially modulates mesolimbic activity states and bivalent affective processing via interactions with the opioid receptor system. We identify a double-dissociation directly in the anterior vs. posterior regions of the NASh capable of producing divergent effects on affective valence processing, MSN neuronal activity and oscillatory patterns, and distinct and dissociable opioid receptor signaling mechanisms regulating reward vs. aversion-related behaviors. Together, these findings have important implications for understanding how the effects of THC in anatomically distinct regions of the NASh may underlie the neuropsychiatric side-effects of cannabis, including its dependence-producing properties and psychotomimetic side-effects.

## References

[1] Parker LA, Gillies T (1995). THC-induced place and taste aversions in Lewis and Sprague-Dawley rats. Behav. Neurosci..

[2] Lepore M, Vorel SR, Lowinson J, Gardner EL (1995). Conditioned Place Preference Induced by delta-9-Tetrahydrocannabinol: Comparison with Cocaine, Morphine, and Food Reward. Life sciences.

[3] Chen J, Paredes W, Lowinson JH, Gardner EL (1991). Strain-specific facilitation of dopamine efflux by ∆9-tetrahydrocannabinol in the nucleus accumbens of rat: An *in vivo* microdialysis study. Neurosci. Lett..

[4] Pacheco-Colón I, Limia JM, Gonzalez R (2018). Nonacute effects of cannabis use on motivation and reward sensitivity in humans: A systematic review. Psychol. Addict. Behav..

[5] Fitoussi A, Zunder J, Han T, Laviolette SR (2018). Delta-9-Tetrahydrocannabinol Potentiates Fear Memory Salience Through Functional Modulation of Mesolimbic Dopaminergic Activity States. Eur. J. Neurosci..

[6] French ED (1997). Delta-9-THC excites rat VTA dopamine neuroons through activation of cannabinoid but not opioid receptors. Neurosci. Lett..

[7] Filbey FM (2016). fMRI study of neural sensitization to hedonic stimuli in long-term, daily cannabis users. Hum. Brain Mapp..

[8] Chen J (1990). Delta-9-Tetrahydrocannabinol produces naloxone-blockable enhancement of presynaptic basal dopamine efflux in nucleus accumbens of conscious, freely-moving rats as measured by intracerebral microdialysis. Psychopharmacology (Berl)..

[9] Hoffman AF, Oz M, Caulder T, Lupica CR (2003). Functional tolerance and blockade of long-term depression at synapses in the nucleus accumbens after chronic cannabinoid exposure. J. Neurosci..

[10] Saddoris MP, Cacciapaglia F, Wightman RM, Carelli RM (2015). Differential Dopamine Release Dynamics in the Nucleus Accumbens Core and Shell Reveal Complementary Signals for Error Prediction and Incentive Motivation. J. Neurosci..

[11] Baliki MN (2013). Parceling Human Accumbens into Putative Core and Shell Dissociates Encoding of Values for Reward and Pain. J. Neurosci..

[12] Bozarth MA (2015). Pleasure Systems in the Brain. Neuron.

[13] Calipari ES (2016). *In vivo* imaging identifies temporal signature of D1 and D2 medium spiny neurons in cocaine reward. Proc. Natl. Acad. Sci..

[14] Arvidsson U (1995). Distribution and targeting of a mu-opioid receptor (MOR1) in brain and spinal cord. J. Neurosci..

[15] Castro DC, Berridge KC (2014). Opioid hedonic hotspot in nucleus accumbens shell: mu, delta, and kappa maps for enhancement of sweetness ‘liking’ and ‘wanting’. J. Neurosci..

[16] Pickel VM, Chan J, Kash TL, Rodríguez JJ, Mackie K (2004). Compartment-specific localization of cannabinoid 1 (CB1) and mu-opioid receptors in rat nucleus accumbens. Neuroscience.

[17] Carlezon WA, Thomas MJ (2009). Biological substrates of reward and aversion: A nucleus accumbens activity hypothesis. Neuropharmacology.

[18] Ahmad T, Sun N, Lyons D, Laviolette SR (2017). Bi-directional cannabinoid signalling in the basolateral amygdala controls rewarding and aversive emotional processing via functional regulation of the nucleus accumbens. Addict. Biol..

[19] Kalenscher T, Lansink CS, Lankelma JV, Pennartz CMA (2010). Reward-Associated Gamma Oscillations in Ventral Striatum Are Regionally Differentiated and Modulate Local Firing Activity. J. Neurophysiol..

[20] Sun Y (2011). Gamma oscillations in schizophrenia: Mechanisms and clinical significance. Brain Res..

[21] Paxinos, G. & Watson, C. *The Rat Brain in Stereotaxic Coordinates*. (Elsevier, 2005). 10.1016/0165-0270(80)90021-7 6110810

[22] Ahmad T, Lauzon NM, de Jaeger X, Laviolette SR (2013). Cannabinoid Transmission in the Prelimbic Cortex Bidirectionally Controls Opiate Reward and Aversion Signaling through Dissociable Kappa Versus -Opiate Receptor Dependent Mechanisms. J. Neurosci..

[23] Norris C (2016). Cannabidiol modulates fear memory formation through interactions with serotonergic transmission in the mesolimbic system. Neuropsychopharmacology.

[24] Laviolette, S. R. & van der Kooy, D. Blockade of mesolimbic dopamine transmission dramatically increases sensitivity to the rewarding effects of nicotine in the ventral tegmental area. *Mol*. *Psychiatry* **8**, 50–59, 9 (2003). 10.1038/sj.mp.4001197 12556908

[25] Ahmad T, Laviolette SR (2017). Cannabinoid reward and aversion effects in the posterior ventral tegmental area are mediated through dissociable opiate receptor subtypes and separate amygdalar and accumbal dopamine receptor substrates. Psychopharmacology (Berl)..

[26] Loureiro M, Kramar C, Renard J, Rosen LG, Laviolette SR (2016). Cannabinoid Transmission in the Hippocampus Activates Nucleus Accumbens Neurons and Modulates Reward and Aversion-Related Emotional Salience. Biol. Psychiatry.

[27] Loureiro M, Renard J, Zunder J, Laviolette SR (2015). Hippocampal Cannabinoid Transmission Modulates Dopamine Neuron Activity: Impact on Rewarding Memory Formation and Social Interaction. Neuropsychopharmacology.

[28] Lintas A (2012). Inputs from the basolateral amygdala to the nucleus accumbens shell control opiate reward magnitude via differential dopamine D1 or D2 receptor transmission. Eur. J. Neurosci..

[29] Skelly MJ, Guy EG, Howlett AC, Pratt WE (2010). CB1 receptors modulate the intake of a sweetened-fat diet in response to mu-opioid receptor stimulation of the nucleus accumbens. Pharmacol. Biochem. Behav..

[30] Richard JM, Fields HL (2016). Mu-opioid receptor activation in the medial shell of nucleus accumbens promotes alcohol consumption, self-administration and cue-induced reinstatement. Neuropharmacology.

[31] Morra JT, Glick SD, Cheer JF (2012). Cannabinoid receptors mediate methamphetamine induction of high frequency gamma oscillations in the nucleus accumbens. Neuropharmacology.

[32] Morra JT, Glick SD, Cheer JF (2010). Neural Encoding of Psychomotor Activation in the Nucleus Accumbens Core, But Not the Shell, Requires Cannabinoid Receptor Signaling. J. Neurosci..

[33] Cheer JF (2004). Cannabinoids Enhance Subsecond Dopamine Release in the Nucleus Accumbens of Awake Rats. J. Neurosci..

[34] Oleson EB, Cheer JF (2012). A brain on cannabinoids: The role of dopamine release in reward seeking. Cold Spring Harb. Perspect. Med..

[35] Rigucci S (2018). Cannabis use in early psychosis is associated with reduced glutamate levels in the prefrontal cortex. Psychopharmacology (Berl)..

[36] Pistis M (2002). Delta(9)-tetrahydrocannabinol decreases extracellular GABA and increases extracellular glutamate and dopamine levels in the rat prefrontal cortex: an *in vivo* microdialysis study. Brain Res..

[37] Draycott B (2014). Cannabinoid Transmission in the Prefrontal Cortex Bi-Phasically Controls Emotional Memory Formation via Functional Interactions with the Ventral Tegmental Area. J. Neurosci..

[38] Di Chiara G, Imperato A (1988). Opposite effects of mu and kappa opiate agonists on dopamine release in the nucleus accumbens and in the dorsal caudate of freely moving rats. J Pharmacol Exp Ther.

[39] Ikemoto S, Panksepp J (1999). The role of nucleus accumbens dopamine in motivated behavior: A unifying interpretation with special reference to reward-seeking. Brain Res. Rev..

[40] Di Chiara G (2004). Dopamine and drug addiction: The nucleus accumbens shell connection. Neuropharmacology.

[41] Kuepper R (2010). Does dopamine mediate the psychosis-inducing effects of cannabis? A review and integration of findings across disciplines. Schizophr. Res..

[42] Fadda P (2006). Cannabinoid self-administration increases dopamine release in the nucleus accumbens. Neuroreport.

[43] Cheer JF (2007). Phasic Dopamine Release Evoked by Abused Substances Requires Cannabinoid Receptor Activation. J. Neurosci..

[44] Bossong MG (2015). Further human evidence for striatal dopamine release induced by administration of δ9-tetrahydrocannabinol (THC): Selectivity to limbic striatum. Psychopharmacology (Berl)..

[45] Bossong MG (2013). The endocannabinoid system and emotional processing: A pharmacological fMRI study with {increment}9-tetrahydrocannabinol. Eur. Neuropsychopharmacol..

[46] Englund A (2013). Cannabidiol inhibits THC-elicited paranoid symptoms and hippocampal-dependent memory impairment. J. Psychopharmacol..

[47] Freeman D (2015). How Cannabis Causes Paranoia: Using the Intravenous Administration of Δ9-Tetrahydrocannabinol (THC) to Identify Key Cognitive Mechanisms Leading to Paranoia. Schizophr. Bull..

[48] Childs E, Lutz JA, de Wit H (2017). Dose-related effects of delta-9-THC on emotional responses to acute psychosocial stress. Drug Alcohol Depend..

[49] Zangen A, Solinas M, Ikemoto S, Goldberg SR, Wise RA (2006). Two brain sites for cannabinoid reward. J. Neurosci..

[50] Mahler SV, Smith KS, Berridge KC (2007). Endocannabinoid hedonic hotspot for sensory pleasure: anandamide in nucleus accumbens shell enhances ‘liking’ of a sweet reward. Neuropsychopharmacology.

[51] Zimmer A (2001). Absence of delta -9-tetrahydrocannabinol dysphoric effects in dynorphin-deficient mice. J. Neurosci..

[52] Solinas M (2005). Cannabinoid agonists but not inhibitors of endogenous cannabinoid transport or metabolism enhance the reinforcing efficacy of heroin in rats. Neuropsychopharmacology.

[53] Caillé S, Parsons LH (2006). Cannabinoid modulation of opiate reinforcement through the ventral striatopallidal pathway. Neuropsychopharmacology.

[54] Uhlhaas PJ, Haenschel C, Nikolić D, Singer W (2008). The role of oscillations and synchrony in cortical networks and their putative relevance for the pathophysiology of schizophrenia. Schizophr. Bull..

[55] Cohen MX (2009). Good vibrations: cross-frequency coupling in the human nucleus accumbens during reward processing. J. Cogn. Neurosci..

[56] Ge S (2018). Oscillatory local field potentials of the nucleus accumbens and the anterior limb of the internal capsule in heroin addicts. Clin. Neurophysiol..

[57] van der Meer MAA, Redish AD (2009). Low and high gamma oscillations in rat ventral striatum have distinct relationships to behavior, reward, and spiking activity on a learned spatial decision task. Front. Integr. Neurosci..

[58] Herrmann CS, Demiralp T (2005). Human EEG gamma oscillations in neuropsychiatric disorders. Clin. Neurophysiol..

[59] Nottage JF (2015). Delta-9-tetrahydrocannabinol, neural oscillations above 20 Hz and induced acute psychosis. Psychopharmacology (Berl)..

[60] Cortes-Briones J (2015). Delta-9-THC Disrupts Gamma (γ)-Band Neural Oscillations in Humans. Neuropsychopharmacology.

[61] Skosnik PD, Krishnan GP, Aydt EE, Kuhlenshmidt HA, O’Donnell BF (2006). Psychophysiological evidence of altered neural synchronization in cannabis use: Relationship to schizotypy. Am. J. Psychiatry.

[62] Radhakrishnan R, Wilkinson ST, D’Souza DC (2014). Gone to pot-a review of the association between cannabis and psychosis. Front. Psychiatry.

[63] Kuepper R (2011). Continued cannabis use and risk of incidence and persistence of psychotic symptoms: 10 year follow-up cohort study. BMJ.

[64] Dejean C (2017). Memories of Opiate Withdrawal Emotional States Correlate with Specific Gamma Oscillations in the Nucleus Accumbens. Neuropsychopharmacology.

